# Heart Failure in Focus: Acute Presentation, Risk Factors and Integration of Artificial Intelligence

**DOI:** 10.3390/jcdd13090415

**Published:** 2026-08-26

**Authors:** Imo A. Ebong, Edidiong I. Akpabio

**Affiliations:** 1 Department of Internal Medicine, Division of Cardiovascular Medicine, University of California Davis, 4860 Y Street, Suite 2860, Sacramento, CA 95817, USA; 2 School of Medicine, Meharry Medical College, Nashville, TN 37208, USA

Heart failure (HF) is a complex syndrome that consists of cardinal clinical symptoms in the context of distinct physical signs, and it is frequently accompanied by a plethora of comorbidities [1]. Epidemiologic trends indicate that HF is an escalating global health challenge that is driven by demographic shifts towards aging populations and a rising prevalence of risk factors [2]. In the United States (US), it is projected that HF will affect 11.4 million people by 2050, with healthcare-related expenditures that could reach $142 billion [3]. This is particularly alarming since HF hospitalization and mortality rates are continuing to increase [3], with marked disparities across geographic regions and demographic groups [2]. This Special Issue in *JCDD*, titled “Epidemiological, Basic Science and Clinical Research Studies in Heart Failure,” offers a broad overview of several issues pertaining to HF, with seven publications covering diverse topics that include acute presentation, risk factors/comorbidities, exercise capacity, the use of artificial intelligence in neuro-modulatory therapy, and considerations for extracorporeal membrane oxygenation (ECMO) use in complex patients presenting with HF-related cardiogenic shock. An illustration of the scope of the contributions published in this Special Issue is presented according to topic area in Figure 1.

In Contribution 1, Boesing et al. validate the performance of known clinical factors for predicting poor outcomes in patients with acute decompensated HF (ADHF) using data commonly obtained in the early presentation phase from an indigenous Swiss population. The predictors identified included poor perfusion status, fluid overload, and comorbidities such as chronic kidney disease (CKD). These findings are corroborated in the European Society of Cardiology Heart Failure Long-Term (ESC-HF-LT) Registry but with substantial variation in the occurrence of adverse outcomes according to clinical profiles, systolic blood pressure, congestion, and hypoperfusion phenotype [4]. The findings reported by Chioncel at al. [4] and Boesing et al. in this Special Issue highlight the importance systematically characterizing patients with ADHF using established clinical predictors at their initial presentation. This information can be applied to guide further diagnostic testing, the level of care, and appropriate treatments. Irrespective of how this information is employed, the risk of mortality in ADHF persists throughout the entire hospitalization period and into the first few months after discharge [4]. Therefore, a time course approach that progressively integrates functional abnormalities such as renal dysfunction, circulatory failure, coagulation disorders, liver dysfunction, and inflammatory reactions is necessary to identify patients at high risk of in-hospital deaths [5].

For the sickest patients who present with cardiogenic shock, Chau et al. (Contribution 2) provide an in-depth discussion of a structured approach to daily care whereby veno-arterial extracorporeal membrane oxygenation (VA-ECMO) is used for circulatory support to maintain tissue perfusion. By emphasizing the importance of multidisciplinary coordination and an individualized patient approach, they provide a nuanced perspective on strategies that can be beneficial for optimizing clinical outcomes and reducing ECMO-related complications.

HF prevention programs should focus on optimizing comorbid conditions that increase the risk of new-onset HF and decompensation in patients with pre-existing HF. These comorbidities include hypertension, diabetes, myocardial ischemia, atrial fibrillation, obstructive sleep apnea, CKD, and acute infections. HF and liver disease also frequently co-exist due to complex cardio-hepatic interactions, alongside shared risk factors like alcohol abuse, drugs, inflammation, autoimmunity, and infections [6]. In Contribution 3, Kostev et al. draw attention to alcoholic and non-alcoholic liver cirrhosis as important HF risk factors, focusing particularly on males with alcoholic liver cirrhosis. Their report is supported by evidence from the Epidemiology of Acute Heart Failure in Emergency Departments (EAHFE) and Registro Nacional de Insuficiencia Cardiaca (RICA) multicenter prospective registries [7]. In the aforementioned cohorts, Miro et al. observed that different HF comorbidities contributed variably to HF mortality, but importantly, liver cirrhosis had stronger associations with HF mortality across the entire spectrum of the left ventricular ejection fraction (LVEF) than other comorbidities [7]. Cirrhotic cardiomyopathy complicates the presentation of approximately 50% of patients with liver cirrhosis and is characterized by systolic dysfunction, impaired diastolic relaxation, prolonged isovolumetric relaxation time, and electrophysiological abnormalities [6,8]. From a pathophysiological perspective, cirrhotic cardiomyopathy is driven by a proinflammatory state that leads to cardiomyocyte apoptosis and a shift in the myosin heavy chain isoform from subtype “a” to the weaker “b” isoform [6]. Other pathophysiological pathways, such as neurohormonal activation, endothelial dysfunction, and circulatory disturbances, act synergistically to propagate a hepato-cardio-renal syndrome [8] that results in multiorgan failure. The excessive risk of morbidity and mortality in patients who have both HF and liver cirrhosis underscores the need for interdisciplinary collaboration between HF cardiologists and hepatologists to improve outcomes in this patient population.

Despite novel advances in diagnostic modalities, medical therapies, support devices, and the surgical management of patients with HF, the trends have remained pervasive. Therefore, preventive measures are critical to curb the HF epidemic by targeting established and novel risk factors. In Contribution 4, Kusyn et al. provide a detailed appraisal of behavioral risk factors for HF and potential targets for prevention-focused interventions that can be meticulously applied to modify behavioral risk. Correction of risky behaviors is beneficial for primary and secondary prevention of HF. The American Heart Association’s Life Essential 8 (LE8), which includes 3 behavioral components (smoking status, physical activity, and diet), is inversely associated with HF risk [9]. In the UK Biobank data, individuals with the lowest LE8 scores (i.e., the least healthy) had a 2.67-times (95% CI: 2.42 to 2.94) higher risk of HF when compared to those with the highest LE8 scores (i.e., the healthiest) after 10.4 years of follow-up [10]. The strength of the association was strongest for individuals under 50 years old, women, and minorities [10]. More importantly, improvements in LE8 scores were beneficial for preventing HF development [9,11]. This implies that the modification of risk factors is crucial for early prevention in order to reduce HF prevalence [12]. The evidence on novel behavioral risk factors such as electronic nicotine delivery systems, cannabis, high-dose caffeine, and psychostimulants is lacking and should be explored further in future studies.

HF can be categorized as HFrEF, HFpEF, or HF with mildly reduced ejection fraction (HFmrEF) when the left ventricular ejection fraction (LVEF) is 40% or less, 50% or greater, and between 41% and 49%, respectively. Although the risk factors overlap, guideline-directed medical therapies (GDMT) that have been successfully used in HFrEF management are not all beneficial in HFpEF, which currently has fewer evidence-based therapies in comparison to HFrEF. The spotlight was directed to cardiometabolic-HFpEF (CM-HFpEF) by Yang et al. in Contribution 5, where the authors provided an in-depth discussion of CM-HFpEF that included the disease-modifying implications of diverse lifestyle interventions. CM-HFpEF, the most common presenting HFpEF phenotype, is a sequelae of metabolic abnormalities triggered by the secretion of a dysfunctional adipokine profile from excess visceral fat, as well as age-related inflammation [13]. This HFpEF phenotype is characterized by immune dysregulation, oxidative stress, lipotoxic damage, microvascular dysfunction, and eventually epigenetic remodeling that impairs myocardial relaxation and diastolic function [14,15]. In the associations of LE8 and HF previously described, the strongest risk reduction derived from improved LE8 scores was seen among patients with HFpEF [16]. In CM-HFpEF, aerobic exercise training and weight loss by caloric restriction or intermittent fasting was associated with improvements in exercise capacity [14,17]. Other lifestyle interventions such as stress management and healthy sleep habits are potentially beneficial in HFpEF due to their inhibitory effects on sympathetic activity and inflammation [14].

Sex differences have been reported in HF epidemiology, clinical presentation, and outcomes, particularly in HFpEF. In a pooled study that included participants enrolled in the CHARM-Preserved (Candesartan in Heart failure: Assessment of Reduction in Mortality and Morbidity), TOPCAT-Americas (Treatment of Preserved Cardiac Function Heart Failure with an Aldosterone Antagonist Trial), and I-Preserve (Irbesartan in Heart Failure with Preserved Ejection Fraction) trials, females had similar rates of hospitalization and better survival than males, despite worse symptoms, more congestion, and lower quality of life [18]. In Contribution 6, Lorenzo et al. report sex disparities in submaximal exercise capacity among HF patients (both HFrEF and HFpEF) in which females exhibited significantly shorter six-minute walk test (6MWT) distances than males irrespective of prognostic clinical, psychosocial, and frailty-related factors. However, the reported evidence is mixed. In a secondary analysis of the RELAX (PhosphodiesteRasE-5 Inhibition to Improve CLinical Status And EXercise Capacity in Diastolic Heart Failure) and NEAT-HFpEF (Nitrate’s Effect on Activity Tolerance in Heart Failure with Preserved Ejection Fraction) trials, baseline 6MWT distance and HF-related quality of life were similar between the sexes [19]. However, 6MWT distance was associated with quality of life in males but not in females [19]. In a cross-sectional study conducted at a single tertiary care center, women with HFpEF had greater cardiac and extracardiac deficits, such as systolic reserve, diastolic reserve, and peripheral O2 extraction, despite having similar percentage predicted exercise capacity [20].

The benefits of neurohormonal modulation with GDMT such as beta blockers, angiotensin-converting enzyme inhibitors (ACEIs), angiotensin receptor blockers (ARBs), angiotensin receptor–neprilysin inhibitors (ARNIs), and mineralocorticoid receptor antagonists (MRAs), has been firmly established for HFrEF [21]. Neuromodulation or autonomic regulation therapy (ART) with devices that directly target the autonomic nervous system is an area of active investigation in HFpEF and HFrEF [21,22]. Examples of such therapies include cardiac sympathetic denervation (CSD), renal nerve denervation (RND), vagus nerve stimulation (VNS), tragus nerve stimulation (TNS), cardiac contractility modulation (CCM), baroreceptor activation therapy (BAT), and spinal cord stimulation (SCS) therapy [21,22,23]. Most of these therapies are still investigational. However, BAT and CCM are approved by the US food and drug administration (FDA) for managing patients with symptomatic HFrEF despite GDMT. RND is also approved as an adjunctive therapy to lifestyle modifications and medications for patients with HF who have uncontrolled or resistant hypertension. In Contribution 7, Ansari et al. provide an overview of the evolving role of artificial intelligence (AI) in neuromodulation therapy for patients with HFrEF and HFpEF. AI can utilize deep phenotyping to improve patient selection, provide instantaneous optimization of vagal nerve stimulation algorithms, and generate closed-loop systems that adapt to patient needs and physiological responses. The large-scale integration of the deep learning components of AI such as artificial neural networks (ANN) and convolutional neural networks (CNN) into HF diagnostic algorithms in conjunction with remote patient monitoring technology through the Internet of Things and mHealth has the potential to significantly impact HF care [24]. Because of their ability to integrate and synthesize large amounts of data with multidimensional interactions, AI-based algorithms could provide physicians with timely information that enables them make decisions efficiently [25]. However, significant barriers such as data privacy concerns, integration challenges, uncertainty about the performance of the AI model in the real world, model governance issues, distrust in AI, and concerns about fairness and bias have limited the clinical uptake of AI into HF practice [26]. Additionally, there are few randomized controlled trials validating the efficacy of AI algorithms in HF care [26]. In the US, the FDA has emphasized the importance of prospective validation of AI algorithms before their implementation in clinical practice [25]. Novel strategies are needed to successfully manage the global HF epidemic. This requires a holistic approach to care that concurrently addresses HF diagnostics, therapeutics, and prevention while providing accommodation for marginalized populations and adverse social determinants of health (SDOH). This Special Issue has provided insight into several facets of the HF conundrum by tackling aspects of acute HF management, targets of HF prevention, sex-based considerations in the physical functioning of HF patients, and potential AI applicability in the neuro-modulatory therapy of HF patients. While the topics covered are not exhaustive, the evidence presented suggests that future research is needed, particularly in areas where the data are still emerging, such as device-based neuromodulation and the application of AI in HF care protocols.

## Figures and Tables

**Figure 1:**
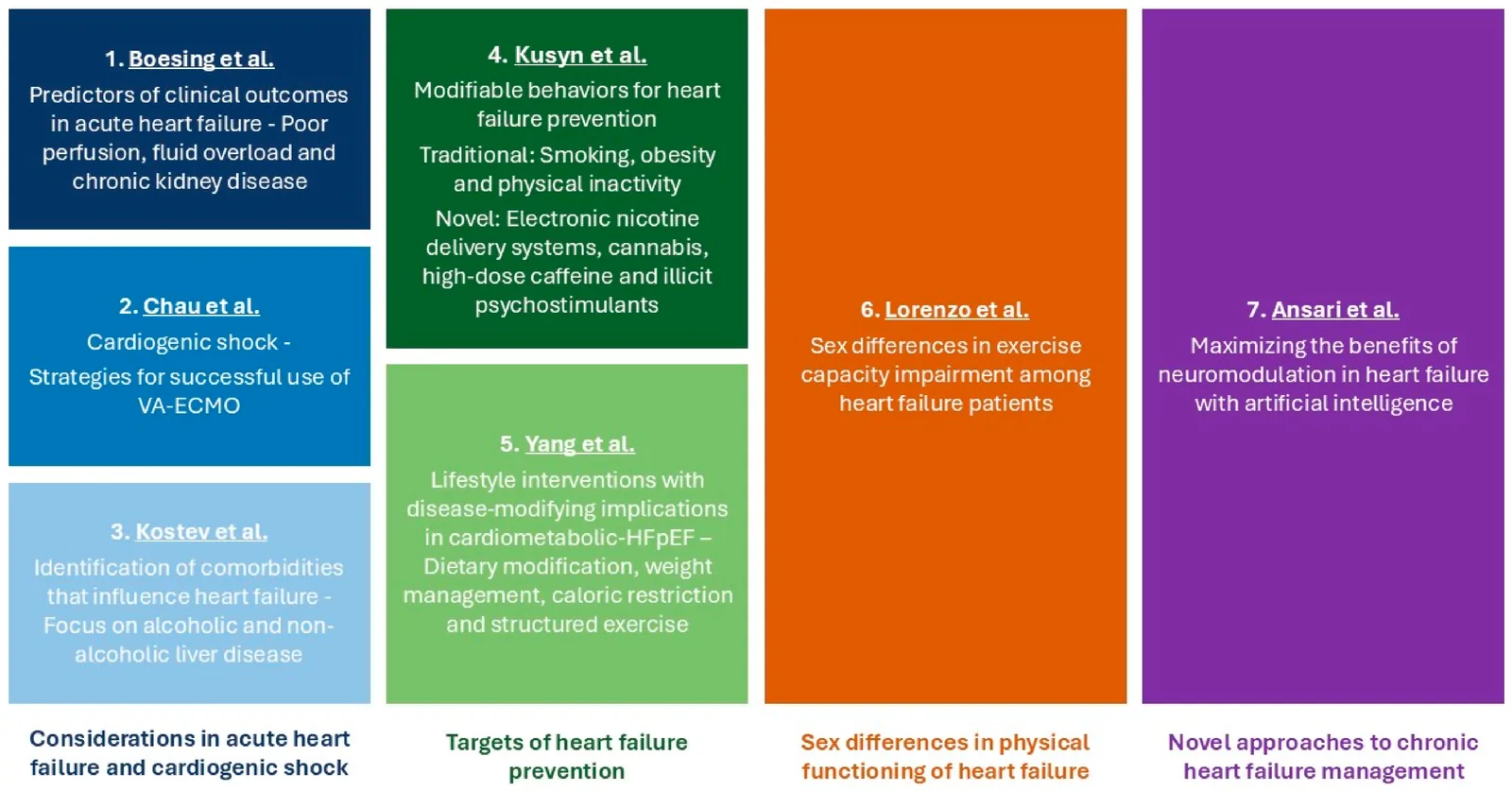
Scope of the contributions in this Special Issue.

## Abbreviations

HFpEF	heart failure with preserved ejection fraction
VA-ECMO	veno-arterial extracorporeal membrane oxygenation
